# Rifampicin efficacy against doxorubicin-induced cardiotoxicity in mice

**DOI:** 10.1186/s43044-023-00403-z

**Published:** 2023-08-21

**Authors:** Omnia A. Basal, Rasha F. Zahran, Entsar A. Saad

**Affiliations:** Chemistry Department, Faculty of Science, Damietta University, Damietta, 34517 Egypt

**Keywords:** Atrial natriuretic peptide, Cardiac troponin, Histopathology, Minerals, Oxidative stress, Vascular endothelial growth factor

## Abstract

**Background:**

The toxic effect of doxorubicin on the heart limits its clinical usage in cancer therapy. This work intended to investigate, for the first time, the efficacy of rifampicin administration against doxorubicin-induction of cardiotoxicity in mice. Forty adult male albino mice were distributed into four sets: Control, Doxorubicin, Doxorubicin + Rifampicin 0.107, and Doxorubicin + Rifampicin 0.214, with n = 10 for each. Heart histopathology and biochemical assays for heart function tests [creatine kinase (CK), lactate dehydrogenase (LDH), aspartate aminotransferase (AST), cardiac troponin I (cTnI), atrial natriuretic peptide (ANP), and vascular endothelial growth factor (VEGF)], oxidative stress [malondialdehyde (MDA) and superoxide dismutase (SOD)], and minerals [phosphorus, sodium, potassium, and calcium] were done.

**Results:**

Doxorubicin-induced cardiotoxicity using a total dose of 15 mg/kg was confirmed histologically. Cardiomyocytes showed congestion, necrosis, edema, and inflammatory cell infiltration. Biochemically, elevations in LDH, CK, and AST activities, *p *< 0.001, as well as increases in cTnI and ANP levels, *p *< 0.001, increased oxidative stress (MDA, *p *< 0.001), high minerals (Na, K, *p *< 0.001, P, *p *< 0.01, and Ca, *p *< 0.05), with reduced VEGF concentration, *p *< 0.001, and low antioxidant (SOD, *p *< 0.001) were observed in the Doxorubicin group compared to control. Co-treatment with rifampicin significantly (*p *< 0.001) reduced the increased oxidative stress, high Na and K, increased LDH, CK, AST, cTnI, and ANP, and elevated the low SOD toward the normal ranges. Our histological data supported our biochemical data; rifampicin dose 0.214 mg/kg showed better improvements than dose 0107.

**Conclusions:**

Our results demonstrated that rifampicin could help protect the body against doxorubicin-induced cardiotoxicity through its antioxidative effect.

## Background

Cardiovascular diseases are the chief cause of death globally; they were responsible for 32% of all deaths in the globe in 2019. Approximately 75% of deaths were in low- and middle-income nations [1, 2]. Heart failure is a widespread, high-cost, and debilitating syndrome that is linked to a much complex drug strategy, a high number of comorbidities, and a large diverse number of healthcare providers [3]. Cardiomyopathy is a heart muscle disorder characterized by structural/or functional alterations that negatively affect how the heart pumps blood. Cardiomyopathies frequently drive to cardiovascular death or advanced-heart failure-related dysfunction [4]. Many regularly used drugs, for example, certain tumor chemotherapeutics, anthracycline-Doxorubicin, cisplatin, the antiretroviral compound azidothymidine, varied oral anti-diabetics, and others also various materials of abuse like alcohol, cocaine, etc. might induce direct cardiotoxicity [5].

Doxorubicin is an anthracyclines antibiotic from *Streptococcus peucetius var. caesius*. It is commercially known as Adriamycin, Doxil, Rubex, widely utilized as a chemotherapeutic to treat various types of solid tumors. However, the doxorubicin drug's severe side effects, such as cardiotoxicity, hepatotoxicity, nephrotoxicity, and neurotoxicity, limit its clinical use [6]. Cardiomyopathy induced by doxorubicin may cause heart failure [7]. An approximate occurrence of 3–5% of heart failure is shown if a cumulative dose of 400 mg doxorubicin/m^2^ is used [8]. The mechanism of how doxorubicin-cardiotoxicity can develop has been studied widely. Nevertheless, no effective proven therapy for doxorubicin-cardiotoxicity is in use [9]. Therefore, looking for a new effective drug is of great interest.

Rifamycins are a class of antibiotics synthesized by *Amycolatopsis rifamycinica* bacteria or artificially. Rifampicin or Rifampin is one of the rifamycin class drugs. It is a semisynthetic antibiotic (commercially known as Rifactine, Rifadin) clinically used to treat bacterial infections like tuberculosis and leprosy. It also aids in curing pruritus in long-standing cholestatic liver disease patients. Its antibacterial action is due to suppressing transcription. It was also tested as an anticancer in vitro and in vivo, with its anti-angiogenic criteria contributing to its anticancer effect [10, 11]. Moreover, its in vitro and in vivo antioxidant effects were tested and established [12]. Rifampicin is also a well-identified activator of the pregnane X receptor, which regulates the genes that play roles in xenobiotics detoxification and elimination [12, 13]. It has been elucidated that it acts efficiently as a protector of the cells, e.g., the liver [12] and the brain [14, 15], against cellular oxidative stress. As some drugs' efficacy sometimes relays on their toxicity in addition to their protective effects. This led us to the thought that rifampicin as an antioxidant may protect against doxorubicin-induced cardiotoxicity.

Therefore, the current study was intended to evaluate, for the first time, the impact of rifampicin on doxorubicin-induced cardiotoxicity in mice.

## Methods

### Chemicals

Doxorubicin (2 mg/mL) [Hikma Specialized Pharmaceuticals, Cairo] and Rifactine (300 mg rifampicin) [MUP (Medical Union Pharmaceuticals), Giza, Cairo, Egypt] were used. Meanwhile, nitro blue tetrazolium (≥ 90%), nicotinamide adenine dinucleotide reduced form (NADH, ≥ 97%), sodium pyrophosphate tetrabasic (≥ 95%), phenazine methosulfate (≥ 90%), trichloroacetic acid (≥ 99%), and thiobarbituric acid (≥ 98%) were obtained from Sigma-Aldrich, Saint Louis, MO, United States. Perchloric acid (70%) was bought from LOBA CHEMIE PVT.LTD, Mumbai, Maharashtra, India. Absolute ethanol (99.9%) was acquired from Alfa Chemical Group, Hadayek El-Kobba, Cairo, Egypt. In addition, potassium dihydrogen orthophosphate (98–100%) and sodium chloride saline solution (0.9%) were purchased from ELNASR PHARMACEUTICAL CHEMICALS, Qalyubia, Egypt. Di-potassium hydrogen phosphate (99%) was bought from HiMedia Laboratories C40, Maharashtra, India. All other solvents and chemicals obtained for the experiments were of the highest available purity and were used as received.

### Experimental animals

Forty adult male Swiss albino mice (body weight of 20–25 g) obtained from Theodor Bilharz Research Institute, Giza, Egypt, were used. Mice were housed at a temperature of 23 ± 2 °C, appropriate humidity, and 12:12 h light/dark cycle following the standards summed up in the “Guide for the Care and Use of Laboratory Animals” designed by the National Academy of Science and published by the National Institute of Health. Mice were accommodated for one week prior using in the experiment. They were kept in clean polypropylene cages and fed with standard mice pellet diet and water ad libitum.

### Induction of cardiotoxicity

To induce cardiotoxicity in mice, a cumulative dose of 15 mg doxorubicin/kg was used [16, 17] as follows: from the stock solution of doxorubicin with a concentration of 2 mg/mL in saline, mice intraperitoneally received a doxorubicin dose of 2.5 mL/kg/day (equivalent to 5 mg/kg/day) diluted directly before injection in saline (10%, v/v) on days 1, 5, and 9 of the experiment.

### Rifampicin doses

Because rifampicin is an already-approved drug used in clinics to treat patients with doses up to 300–600 mg/day (5–10 mg/kg). Moreover, because drug adverse events are dose-related and for safety purposes, two rifampicin doses equal to 0.107 [11] and 0.214 mg/kg, less than 1/20th of the lowest clinically applied dose (5 mg/kg), were selected for studying the efficacy of rifampicin administration against doxorubicin-induction of cardiotoxicity. By choosing very low doses, we expect no/minimal adverse effects.

Practically, rifampicin powder was first dissolved as a stock solution of 1 mM concentration in absolute ethanol, and the volume corresponding to 0.107 mg/kg or 0.214 mg/kg was diluted in saline (10%, v/v) just before injection.

### Experimental groups

Forty mice were divided into four groups, each containing ten mice, and treated as follows (Table 1):

- *Control* (negative control): normal healthy mice
- *Doxorubicin* (cardiotoxicity, positive control): doxorubicin 5 mg/kg (three doses); i.p. on the 1st, 5th, and 9th day
- *Doxorubicin* + *Rifampicin 0.107* (first treated cardiotoxicity group with rifampicin): doxorubicin 5 mg/kg (three doses); i.p. on the 1st, 5th, and 9th day + rifampicin 0.107 mg/kg/day; i.p., for 14 days
- *Doxorubicin* + *Rifampicin 0.214* (the second treated cardiotoxicity group with rifampicin): doxorubicin 5  mg/kg (three doses); i.p. on the 1st, 5th, and 9th day + rifampicin 0.214 mg/kg/day; i.p., for 14 days.

**Table 1:** Experimental design, timeline for drug administration, and end of experiment in different groups

Group name	Description	Drug treatment	Dose (mg/kg/day)	Refs.	Frequency	Exp. days of injection	Route	Exp. duration	Day of scarification
Control	Negative control	–	–	–	–	–	–	14 days	14
Doxorubicin	Positive control (cardiotoxicity)	Doxorubicin	5	[16, 17]	3 times (4 day intervals)	1,5,9	i.p	14 days	14
Doxorubicin + Rifampicin 0.107	First treated group with rifampicin	Doxorubicin	5	[16, 17]	3 times (4 day intervals)	1,5,9	i.p	14 days	14
		Rifampicin	0.107	[11]	Once daily	1–14	i.p		
Doxorubicin + Rifampicin 0.214	Second treated group with rifampicin	Doxorubicin	5	[16, 17]	3 times (4 day intervals)	1,5,9	i.p	14 days	14
		Rifampicin	0.214	–	Once daily	1–14	i.p		

Doxorubicin stock solution (2 mg/mL) was diluted in saline (10%, v/v) before injection whereas rifampicin was prepared as a stock solution of 1 mM concentration in absolute ethanol and it was diluted also in saline (10%, v/v) before injection

*i.p.* Intraperitoneal, *Ref.* Reference, *n* 10 mice in each group

After the last drug injection on the 14th day, the mice fasted for eight hours before being euthanized by cervical dislocation while under general anesthesia. The sacrifice was carried out following the Guide for the Care and Use of Laboratory Animals and in compliance with the ARRIVE guidelines, also approved by our Faculty. Blood samples, just before euthanasia, were collected via cardiac puncture under the effect of combined ketamine (75 mg/kg)/xylazine (10 mg/kg) anesthesia. After clotting, blood was centrifuged. The obtained serum was stored at − 20 °C till use. Rapidly, the heart was separated, washed, dried on filter papers, and weighed. A part of the tissue was kept in 10% buffered formalin for processing for histopathological examination. A 10% W/V tissue homogenate in ice-cold PBS was prepared, centrifuged, and the clear supernatant was stored at − 20 °C till use.

### Biochemical assays

Estimations of phosphorus, sodium, potassium, calcium, CK, LDH, and AST in serum, besides assays of cTn I, ANP, and VEGF levels in tissue homogenate, were performed following kit manufacturer instructions.

The colorimetric kit for LDH assay was purchased from Chema Diagnostica, Italy. Using LDH, pyruvate is converted into L-lactate and NADH into NAD^+^. The rate of NADH/NAD^+^ conversion was followed at 340 nm, and it was proportional to LDH activity [18].

Mouse ANP and Rat VEGF-A (Vascular Endothelial Cell Growth Factor A) immunoassay kits that use the Sandwich-ELISA principle [19] were bought from Ellabscience Biotechnology (USA). Briefly, for VEGF-A, 100 μL of standard or sample were added to the plate wells, incubated for 90 min at 37 °C, 100 μL of Biotinylated Detection Antibody were added, incubated for 60 min at 37 °C, and the plate was washed. For ANP, 50 μL of standard or sample were added to the wells, immediately 50 μL of Biotinylated Detection Antibody were added, incubated for 45 min at 37 °C, and the plate was washed. Then, for both VEGF-A and ANP, 100 μL of horseradish peroxidase (HRP) conjugate were added, incubated for 30 min at 37 °C, the wells were washed, 90 μL of Substrate Reagent were added, incubated for 15 min at 37 °C, 50 μL of Stop Solution were added, and the absorbance was read at 450 nm.

Colorimetrically, CK and AST activities were measured using the N-acetyl-cystein-activated CK and AST kits obtained from Biomed Diagnostics, Germany. The reaction of amino group transfer from aspartate to α-ketoglutarate, producing glutamate and oxaloacetate, was catalyzed by AST. AST activity was proportional to the amount of the formed oxaloacetate, which was measured by reacting with 2,4-dinitrophenylhydrazine in an alkaline solution at 505 nm [20]. For CK, it converted creatine phosphate and adenosine diphosphate to creatine and adenosine triphosphate. Hexokinase converted ATP and glucose into ADP and glucose-6-phosphate, and then glucose-6-phosphate dehydrogenase oxidized the glucose-6-phosphate with the reduction of nicotinamide adenine dinucleotide phosphate. The rate of formation of the nicotinamide adenine dinucleotide phosphate reduced form measured at 340 nm was proportional to CK activity [21].

The Finecare TM cTn I Quantitative kit based on fluorescence immunoassay technology (Finecare TM, Guangzhou Wondfo Biotech, Lizhishan Road, China) was intended to assess cTn I. By adding the specimen to the sample well of the test cartridge, the fluorescence-labeled detector cTn I antibody bound to the cTn I antigen in the sample. When the sample mixture migrated on the nitrocellulose matrix of the test strip via capillary action, the complexes of the detector antibody and cTn I were caught by the cTn I antibody that had been immobilized on the test strip. Consequently, the more cTn I antigen is in the sample, the more complexes were accumulated on the strip. The signal intensity of fluorescence of the detector antibody reflected the amount of cTn I captured, and the Finecare TM FIA system showed cTn I concentrations in the sample [22].

Kits for colorimetric determination of calcium, phosphorus, sodium, and potassium were obtained from Biodiagnostics (diagnostic and research reagents), Giza, Egypt. In short, Ca^2+^ ions were reacted in an alkaline medium with methyl thymol blue, producing a blue color that was measured at 585 nm [23]. For phosphorous, it was reacted with molybdic acid forming phosphomolybdate, which was turned blue via reduction by stannous chloride. The blue color was measured at 640 nm [24]. About sodium level, in excess uranyl acetate, Na^+^ ions with magnesium acetate were converted into sodium magnesium uranyl acetate, and the residual uranyl acetate was reacted with potassium ferrocyanide to form a colored complex that was measured at 545 nm [25]. While potassium ions were reacted with sodium tetraphenyl boron, forming a colloidal solution absorbed at 420 nm [26].

SOD activity in tissue was assayed by the method of Dechatelet et al. [27] depending on the SOD ability to inhibit the phenazine methosulfate -mediated reduction of nitro blue tetrazolium dye. Briefly, 0.1 mL of the homogenate was added to 2.8 mL of a mixture of nitro blue tetrazolium and NADH in pyrophosphate buffer, pH 8.3. Immediately after adding 0.1 mL of phenazine methosulfate, the absorbance at 560 nm was followed for 5 min, and the change per minute was calculated. The % inhibition of color progress was determined depending on that of a control tube. The amount of tissue MDA was measured by the thiobarbituric acid assay [28], which is based on MDA reaction with thiobarbituric acid to give a red color absorbed at 535 nm. In short, 0.2 mL of homogenate was added to a mix of sodium dodecyl sulfate, acetic acid, and thiobarbituric acid. The mixture was heated at 95 °C for 60 min and cooled, then 1 mL of distilled water and 5 mL of the mix of n-butanol and pyridine were added and vigorously shaken. After centrifugation, the organic layer was taken, and its absorbance was read at 535 nm.

### Histopathology

Routinely, heart tissues were fixed in 10% buffered formalin. After dehydration by passing in an ascending series of alcohol and clearance with xylene, fixed tissue was immersed in paraffin wax, sliced into 5–6 μm sections in a rotary microtome, then stained with the nuclear stain hematoxylin and the counterstain eosin (H&E). Under light microscopy, a pathologist unaware of the mice treatment examined and evaluated the stained sections. At least 3-sections were examined by the pathologist per one tissue sample. The magnification X 200 was used. The assessed variables include the evaluation of the parameters of myocardial necrosis (necrosis, infiltration, and hemorrhage), inflammation, and fibrosis.

### Statistical analysis

For comparison between the means of more than two groups, the statistical analysis by the one-way analysis of variance (ANOVA) for parameters with Gaussian distribution was done using the Instat software, version 3.10 (GraphPad, Inc., Sorrento Valley, San Diego, USA), followed by Tukey-Karmer multiple-comparisons test. For data with non-Gaussian distribution Kruskal–Wallis test was employed, followed by Dunnett’s test for multiple comparisons. Data was presented as mean ± standard deviation (S.D). *P *< 0.05 was assumed significant.

## Results

### Effect on heart weight (HW) and heart weight/body weight (HW/BW) ratio

**Fig. 1:**
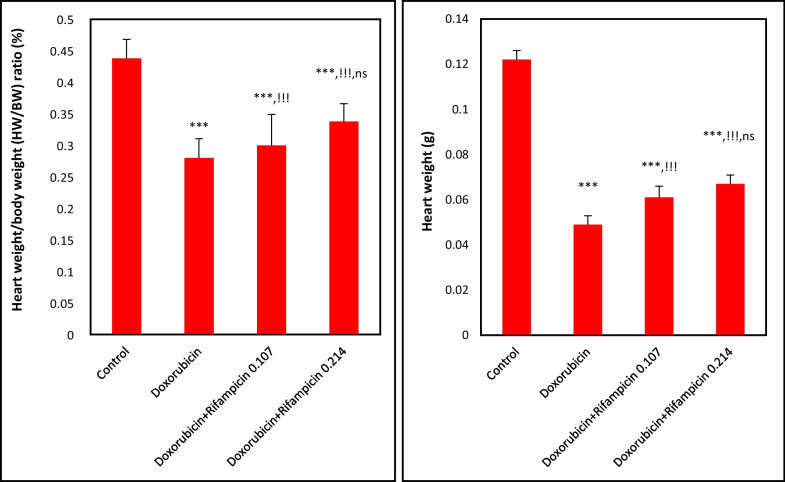
The heart weight (HW) and heart weight/body weight (HW/BW) ratio on the day of scarification for all mice groups. Results are expressed as mean ± S.D, n = 10 mice in each group. ***: *p *< 0.001 versus Control group. !!!: *p *< 0.001 versus Doxorubicin group. ns: *p *> 0.05 versus Doxorubicin + Rifampicin 0.107 group

In Fig. 1, the injection of doxorubicin significantly (*p *< 0.001) resulted in approximately a 60% reduction in HW and reduced HW/BW ratio to 64% of the Control group value; as the mean values of HW and HW/BW ratio were of 0.122 g and 0.438% in the Control group and 0.0489 and 0.28% in the Doxorubicin group. In the concomitant treatment of rifampicin with doxorubicin, with rising the rifampicin dose mean levels of HW and HW/BW were significantly (*p *< 0.001) increased to reach 0.061 and 0.067 g, and 0.3 and 0.34% with the 0.107 mg/kg dose and the 0.214 mg/kg dose, respectively compared to doxorubicin alone. However, by comparing the 0.107 and 0.214 mg/kg doses together, the differences in HW or HW/BW ratio were non-significant.

### Effect on enzymes CK, LDH, and AST

**Fig. 2:**
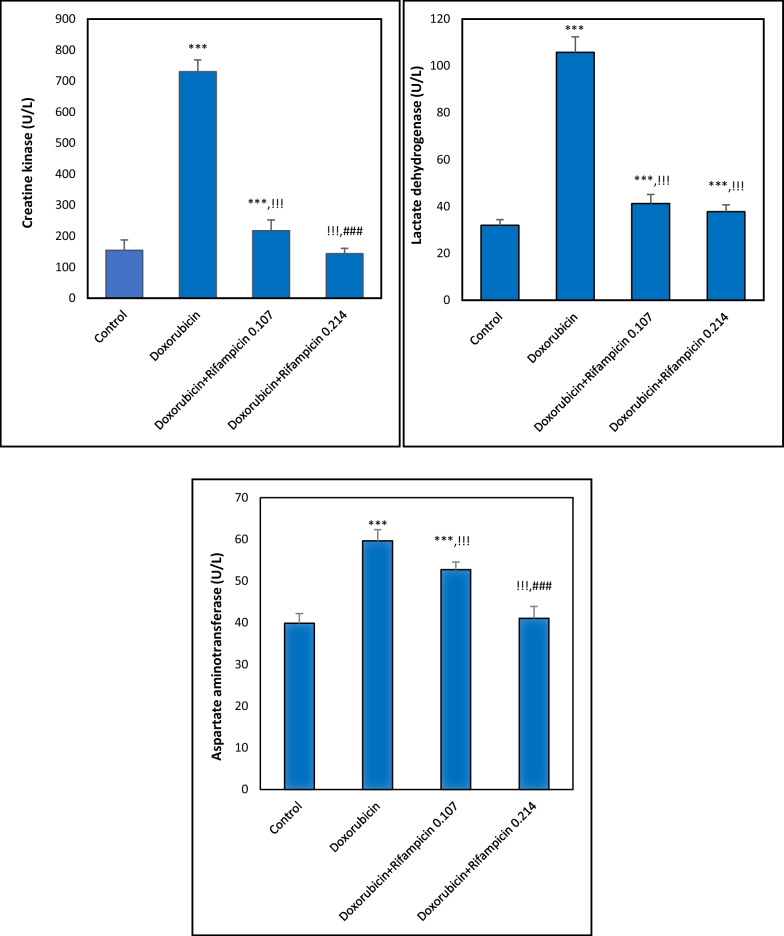
Creatine kinase (CK), lactate dehydrogenase (LDH), and aspartate aminotransferase (AST), activities in all mice groups. Results are expressed as mean ± S.D, n = 10 mice in each group. ***: *p *< 0.001 versus Control group. !!!: *p *< 0.001 versus Doxorubicin group. ###: *p *< 0.001 versus Doxorubicin + Rifampicin 0.107 group

As illustrated in Fig. 2, there were increases in CK, LDH, and AST mean activities (730.39, 105.82, and 59.65 U/L, respectively) to approximately 472%, 331%, and 150% in the Doxorubicin group compared to the Control group (154.69, 32, and 39.86 U/L, respectively, *p *< 0.001). Compared with the Doxorubicin group, in Rifampicin-treated groups (Doxorubicin + Rifampicin 0.107 and Doxorubicin + Rifampicin 0.214), the mean levels of these enzymes were significantly (*p *< 0.001) decreased, to 217.45, 41.3, and 52.71 U/L, respectively in Doxorubicin + Rifampicin 0.107 and to 143.62, 37.8, 41.08 U/L, respectively in Doxorubicin + Rifampicin 0.214, toward normalization with increasing the dose of rifampicin. Statistically, when Rifampicin-treated groups were compared together, differences were significant (*p *< 0.001) in the levels of CK and AST but non-significant in the LDH level.

### Effect on cTn I, ANP, and VEGF

**Fig. 3:**
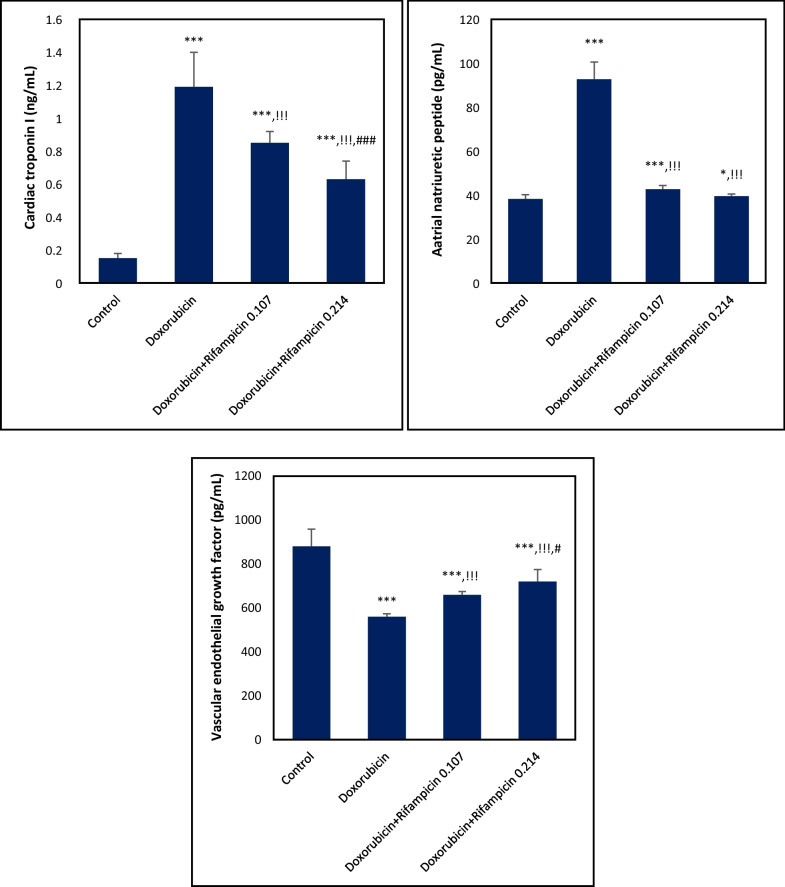
Cardiac troponin I (cTn I), atrial natriuretic peptide (ANP), and vascular endothelial growth factor (VEGF) levels in all mice groups. Results are expressed as mean ± S.D, n = 10 mice in each group. *, ***: *p *< 0.05, *p *< 0.001, respectively versus Control group. !!!: *p *< 0.001 versus Doxorubicin group. #, ###: *p *< 0.05, *p *< 0.001, respectively versus Doxorubicin + Rifampicin 0.107 group

Similarly, the cTn I mean level elevated to 1.19 ng/mL (793%), and the ANP mean level showed an elevation to 92.74 pg/mL (243%) in the Doxorubicin group compared to those in the Control group (0.15 ng/mL and 38.2 pg/mL, *p *< 0.001). In Doxorubicin + Rifampicin 0.107 and Doxorubicin + Rifampicin 0.214 groups, there were decreases towards normal (0.85, 0.63 ng/mL, and 42.72, 39.51 pg/mL in Doxorubicin + Rifampicin 0.107 and Doxorubicin + Rifampicin 0.214, respectively) compared with the Doxorubicin group in a dose-dependent manner (*p *< 0.001). When the two rifampicin groups were compared with each other, there was a significant difference (*p *< 0.001) in cTn I level but not in the ANP level (*p *> 0.05). On the contrary, compared to the Control group, the VEGF mean level displayed a 36% decrease in the Doxorubicin group (560.01 versus 880.12 pg/mL, *p *< 0.001) and re-increased to 658.64 and 720.58 pg/mL depending on the dose significantly (*p *< 0.001) by rifampicin treatment. Moreover, there was a significant difference (*p *< 0.05) in VEGF level in the Doxorubicin + Rifampicin 0.107 group compared to the Doxorubicin + Rifampicin 0.214 group (Fig. 3).

### Effect on sodium, calcium, phosphorous, and potassium

**Fig. 4:**
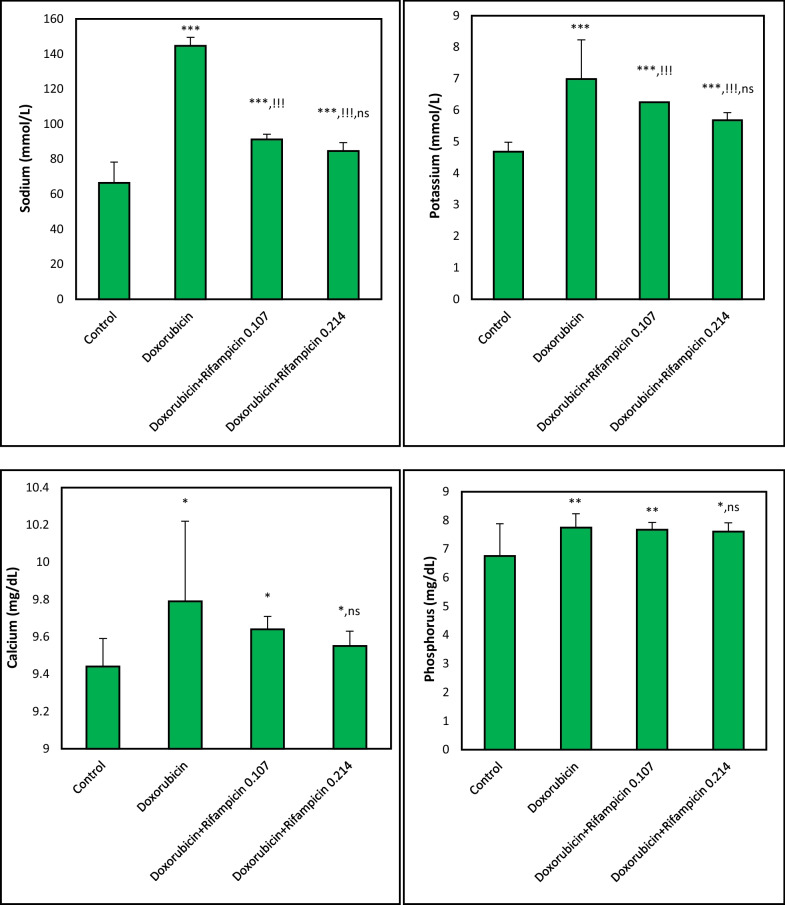
Sodium, potassium, calcium, and phosphorus levels in different mice groups. Results are expressed as mean ± S.D, n = 10 mice in each group. *, **, ***: *p *< 0.05, *p *< 0.01, *p *< 0.001, respectively versus Control group. !!!: *p *< 0.001 versus Doxorubicin group. ns: *p *> 0.05 versus Doxorubicin + Rifampicin 0.107 group

As revealed in Fig. 4, compared to control, mean levels of sodium, calcium, phosphorous, and potassium were significantly (*p *< 0.05–*p *< 0.001) elevated by doxorubicin to 218%, 104%, 115%, and 149%, respectively. They displayed mean levels of 66.39 mmol/L, 9.44 mg/dL, 6.76 mg/dL, and 4.68 mmol/L in the Control group, and 144.67 mmol/L, 9.79 mg/dL, 7.75 mg/dL, and 6.99 mmol/L, respectively in the Doxorubicin group. Rifampicin treatment lowered these levels depending on the dose in Doxorubicin + Rifampicin 0.107 and Doxorubicin + Rifampicin 0.214 groups to levels closer to these in the Control group. However, in the comparison between the Rifampicin-treated groups, all the differences were not significant (*p *> 0.05).

### Effect on oxidative stress

Doxorubicin increased (*p *< 0.001) the MDA mean level to 223%, from 0.048 to 0.107 nmol/g ptn, and decreased (*p *< 0.001) SOD by 51% (from 43.66 to 22.37% inhibition) compared to control. The Doxorubicin + Rifampicin 0.107 and 0.214 groups showed decreases in MDA to 0.06 and 0.056 nmol/g ptn, by 56% and 52%, respectively (*p *< 0.001), alongside elevations in SOD to 37.24 and 36.12% inhibition, by 166% and 161.5%, respectively (*p *< 0.001) compared to the Doxorubicin group. Differences between the Doxorubicin + Rifampicin groups with each other in MDA or SOD levels were statistically non-significant (Table 2).

**Table 2:** Malondialdehyde (MDA) level and superoxide dismutase (SOD) activity in all mice groups

Groups	MDA (nmol/g ptn)	SOD (% inhibition)
Control	0.048 ± 0.006	43.66 ± 4.45
Doxorubicin	0.107 ± 0.01^***^	22.37 ± 1.28^***^
Doxorubicin + Rifampicin 0.107	0.060 ± 0.003^***,!!!^	37.24 ± 1.48^***,!!!^
Doxorubicin + Rifampicin 0.214	0.056 ± 0.003^***,!!!,ns^	36.12 ± 0.76^***,!!!,ns^

Results are expressed as mean ± S.D, n = 10 mice in each group

***P < 0.001 versus Control group

^!!!^*P *< 0.001 versus Doxorubicin group

^ns^*P *> 0.05 versus Doxorubicin + Rifampicin 0.107 group

### Effect on heart pathology

**Fig. 5:**
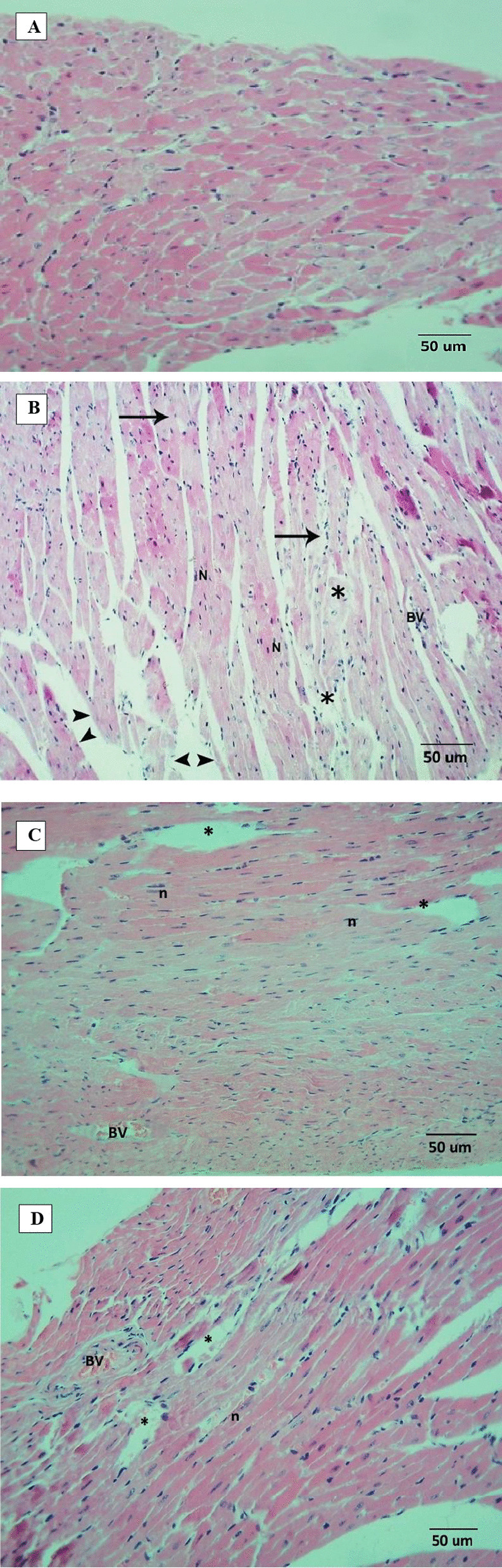
Heart sections histopathology (Hematoxylin and eosin, X 200). **A** Heart section of Control group; section showed normal contact myocardial fibers, clear nuclei, intact cell membrane, and normal cell arrangements. **B** Heart section of Doxorubicin group; section showing severe affection of the myocardium with dispersed myofibrils (Stars) separated by congested blood vessels (BV). The individual fibers are multinucleated (N). There were obvious myocardial cell swelling, widened muscle space of cardiac myocytes and areas of intervening edema, congestion, necrosis (*), inflammatory cell infiltration, and showed myocardial cell damage. Wide spaces are marked by the distance between arrowheads and vacuolated cytoplasm (arrow). **C** Heart section of Doxorubicin + Rifampicin 0.107 group; section showing moderate affection of the cardiac muscle in the form of patches of dispersed myofibrils, large pale area associated with bleeding and areas of intervening edema (Star) and individual fibers nuclear pyknosis (n). **D** Heart section of Doxorubicin + Rifampicin 0.214 group; section showing mild affection of the cardiac muscle in the form of less marked dispersion, edema (Star), less frequent individual fibers nuclear pyknosis (n) with congested blood vessel (BV) and most of myocardial fibers were multinucleated and running parallel to the apical basal axis

After fourteen days of treatment with rifampicin, histological analysis was done for the heart tissues of all groups. The heart of the Control groups had normal morphology and cardiac myofibrils (Fig. 5A). Doxorubicin injection caused myocardial cell toxicity, including cardiomyocyte congestion associated with necrosis, myocardial cell swelling, and intermuscular edema, with inflammatory cell infiltration, vacuolated cytoplasm, dispersed myofibrils separated by congested blood vessels, and myocardial cell damage (Fig. 5B). There was a gradual improvement in toxicity of heart tissues caused by doxorubicin depending on the dose of rifampicin; the heart tissues of Doxorubicin + Rifampicin 0.107 showed moderate affection (Fig. 5C), and the heart tissues of Doxorubicin + Rifampicin 0.214 group showed mild affection (Fig. 5D) as compared to normal heart tissues of Control group.

## Discussion

Doxorubicin antineoplastic drug’s threat to various organs, particularly the heart, has been a motif for researchers to search for solutions in different ways [29, 30]. In the present investigation, doxorubicin-induced cardiac toxicity was confirmed histopathologically; dispersed myofibrils separated by congested blood vessels were observed. The individual fibers appeared multinucleated. There was obvious myocardial cell swelling, widened muscle space of cardiac myocytes, and areas of intervening edema, congestion, necrosis, and inflammatory cell infiltration. These signs of cardiomyocytes damage cause cardiac morbidities, which include changes in myocardial structure and function, as recently proved in Abdelatty et al., Ikewuchi et al., and Ling et al., [31–33]. Physically, doxorubicin injection caused HW and HW/BW ratio decrease as a distinct sign associated with cardiotoxicity. This weight collapse may be due to the mice's declined food intake; we noticed a loss of appetite. This is in agreement with numerous studies that have studied the doxorubicin toxicity on the intestinal mucosa that led to food intake reduction and weight loss [34]. Doxorubicin decreases appetite by indirect toxicity on the gastrointestinal tract by reducing the secretion of internal hormones [35]. Co-treatment with rifampicin stimulated gradual restoration of the HW and HW/BW ratio towards normal; the high dose of rifampicin showed better effect. Rifampicin may improve appetite via increasing leptin, a mediator of energy metabolism. This hypothesis is supported by the study of Mexitalia et al. (2017), who found increases in leptin levels and energy, protein, and fat intake after anti-tuberculosis drugs isoniazid, rifampicin, and pyrazinamide administration. Elevated leptin levels may stimulate appetite resulting in improved dietary intake and increased weight gain [36].

The elevated serum activities of heart damage-related enzymes CK, LDH, and AST in doxorubicin-induced cardiotoxicity could be ascribed to the massive increment in free radical amounts, ascertained by elevated MDA level to more than two-fold, and their effects on cardiomyocytes cellular membrane leading to the outflow of these enzymes from the damaged membranes into the bloodstream as in harmony with [37–40]. Another valuable marker for the evaluation of myocardial damage is cTn I; it plays a role in muscle contraction and is highly sensitive to heart specificity. In agreement with other reports [35, 40–43] we detected elevation in cTn I level after doxorubicin administration that could be linked with cardiomyocytes death [44] might as a result of a massive rise in reactive oxygen species and their effects on cardiomyocytes leading to cellular necrosis, evidenced histopathologically in the heart section of Doxorubicin group, and cardiac insufficiency. Compared to doxorubicin alone, Doxorubicin + Rifampicin displayed dose-dependent decreases in the activities of CK, LDH, and AST to close to the normal and a gradual relief in cTn I levels, indicating that rifampicin can protect against doxorubicin-induced cardiac damage.

For known, VEGF-A plays a crucial role in vascular homeostasis. In the heart, VEGF-A is secreted from various types of cells, including endothelial cells and mature cardiomyocytes. VEGF-A expression increases early after myocardial infarction. In the current study, VEGF-A protein level was reduced due to doxorubicin injection-caused cardiac abnormalities, decreased cells viability, endothelial cell injury, and reduced expression of VEGF-A in the heart, as previously proved in other studies [45, 46]. By treatment with rifampicin (Doxorubicin + Rifampicin), VEGF-A levels were increased with increasing rifampicin dose, suggesting rifampicin can reduce cellular injury.

The natriuretic peptides reflect an early response to cardiac insufficiency. Moreover, the induction of oxidative stress leads to neurohormonal system activation, increasing adrenergic activity, and the release of natriuretic peptides, e.g., ANP [47, 48]. In the same line, in our current work, the rise to 223% in MDA and 51% lowering in SOD confirmed the induction of oxidative stress by doxorubicin administration resulting in increasing the adrenergic activity manifested by elevated ANP level, as in harmony with [49, 50]. The antioxidative and cardio-protective effects of rifampicin were observed; the accompanying injection of rifampicin with doxorubicin stimulated an increase to more than 150% in the antioxidant SOD, a decrease in MDA by more than 50%, and a reduction in the release of ANP. Likewise, Lee et al. [12], demonstrated that rifampicin effectively protects the liver against cellular oxidative stress.

Sodium is a fundamentally functional electrolyte in blood pressure balance. There is an association between hypernatremia and developing heart and kidney diseases, as shown in earlier studies [35, 51] who reported an increase in serum sodium that may be attributable to a lack in urine sodium excretion due to declined sodium reabsorption due to disorders in renal tubules. Moreover, many studies are showing the effect of doxorubicin on serum potassium levels, some demonstrated hypokalaemia [35, 52] but other revealed elevated levels due to the central role of the kidney in the maintenance of potassium homeostasis even in the setting of the chronic renal failure [53]. Although hypokalemia condition is common in heart failure patients, hyperkalemia may appear as a condition of heart failure as a result of many reasons as an implication of neurohormonal alterations in renal disorders and is attached to a higher risk of cardiovascular complications as in harmony with Rakisheva et al. [54]. In our current study, under the influence of doxorubicin, we found high serum sodium and potassium levels due to the effect of doxorubicin on sodium/potassium pumps leading to cardiac dysfunction and cardiomyocyte damage. Also, we found high serum calcium levels, probably due to the ability of doxorubicin to stimulate the release of calcium ions from the endoplasmic reticulum of cardiomyocytes. These calcium ions induce free radicals production that is responsible for oxidative stress and subsequent cardiac dysfunction. This is in consent with many former studies [55–57] who proved the doxorubicin-induced calcium ions imbalance and overload. According to Mitry and Edwards [58], doxorubicin causes loss of calcium homeostasis by affecting the calcium pump found in the membrane and interfering with calcium reservation by the sarcoplasmic reticulum. Likewise, doxorubicin alters the sodium/potassium pump, which affects the sodium gradient required for calcium to flow into the sarcolemma of cardiomyocytes allowing uncontrolled calcium flow out from it. Additionally, doxorubicin stimulates a reduction in the calcium storing amplitude of mitochondria and exacerbates calcium overload. In harmony with Jung et al. [57], serum phosphorus levels showed elevation in our cardiotoxicity model. Hyperphosphatemia may result from doxorubicin's indirect toxic effects on the kidneys. Deteriorated kidney reduces the renal phosphate elimination rate, so serum phosphate levels elevate [59]. In total, in our model, hypernatremia, hyperkalemia, hypercalcemia, and hyperphosphatemia were common symptoms attributed to ''direct'' toxic effects of doxorubicin on the heart and to the extended ''indirect'' doxorubicin toxic effects on the kidneys. Decreasing in elevated levels of Na, K, P, and Ca was observed in a dose-dependent manner in groups that received rifampicin concomitant with doxorubicin, reflecting rifampicin's positive effect on mineral imbalance.

In our scenario, doxorubicin has numerous toxicity degrees on body organs such as the heart and kidney due to free radicals generation and produced oxidative stress. Biochemical evidence includes elevated serum Na, K, Ca, and P levels, in addition to the high levels of CK, LDH, AST, cTn I, and ANP, along with the decrease in the level of VEGF-A. Co-treatment of doxorubicin with rifampicin showed a dose-dependent cardioprotective effect of rifampicin represented by (1) improved levels of the above biomarkers toward normal concomitant to (2) architectural improvement in the form of moderate and mild affection of the cardiac muscle shown histopathologically with rifampicin dose of 0.107 mg/kg and 0.214 mg/kg, respectively.

A limitation of our work is that due to financial causes, we could not study the effect of a longer duration of rifampicin co-treatment on tissue recovery. Also, we could not assess anti-inflammatory and anti-apoptotic mechanisms. Another limitation is that we could not carry out Echocardiography to assess cardiac function.

## Conclusions

In conclusion, our data demonstrate that rifampicin as antioxidant can help the host body face the manifestations of doxorubicin-induced cardiotoxicity; it achieved success in alleviating oxidative stress, amelioration and retrieving the heart structure and function back close to its healthy state. Therefore, rifampicin co-treatment can protect against doxorubicin-induced cardiotoxicity. Finally, our current results recommend future using of rifampicin in combination with doxorubicin in the course of treating cancer to protect against doxorubicin-induced cardiotoxicity after more validation via future research.

## Data Availability

Applicable upon request.

## Abbreviations

CK: Creatine kinase
LDH: Lactate dehydrogenase
AST: Aspartate aminotransferase
CTnI: Cardiac troponin I
ANP: Atrial natriuretic peptide
VEGF: Vascular endothelial growth factor
MDA: Malondialdehyde
SOD: Superoxide dismutase
i.p.: Intraperitoneal
NADH: Nicotinamide adenine dinucleotide reduced form
ELISA: Enzyme-linked immunosorbent assay
HRP: Horseradish peroxidase
H&E: Hematoxylin and eosin
ANOVA: Analysis of variance
S.D: Standard deviation
HW: Heart weight
HW/BW ratio: Heart weight/body weight ratio
